# Predictive factors of 90-day mortality after curative hepatic resection for hepatocellular carcinoma: a western single-center observational study

**DOI:** 10.1007/s00423-024-03337-5

**Published:** 2024-05-03

**Authors:** Sascha Vaghiri, Nadja Lehwald-Tywuschik, Dimitrios Prassas, Sami Alexander Safi, Sinan Kalmuk, Wolfram Trudo Knoefel, Levent Dizdar, Andrea Alexander

**Affiliations:** 1 Department of Surgery (A), Heinrich-Heine-University and University Hospital Duesseldorf, Duesseldorf, Germany; 2 Department of Surgery, Katholisches Klinikum Essen, Philippusstift, Teaching Hospital of Duisburg-Essen University, Huelsmannstrasse 17, 45355 Essen, Germany

**Keywords:** 90-day mortality, Hepatocellular carcinoma, Curative resection, Predictive factors

## Abstract

**Purpose:**

The aim of this study was to identify predictive risk factors associated with 90-day mortality after hepatic resection (HR) in hepatocellular carcinoma (HCC).

**Methods:**

All patients undergoing elective resection for HCC from a single- institutional and prospectively maintained database were included. Multivariate regression analysis was conducted to identify pre- and intraoperative as well as histopathological predictive factors of 90-day mortality after elective HR.

**Results:**

Between August 2004 and October 2021, 196 patients were enrolled (148 male /48 female). The median age of the study cohort was 68.5 years (range19-84 years). The rate of major hepatectomy (≥ 3 segments) was 43.88%. Multivariate analysis revealed patient age ≥ 70 years [HR 2.798; (95% CI 1.263–6.198); *p* = 0.011], preoperative chronic renal insufficiency [HR 3.673; (95% CI 1.598–8.443); *p* = 0.002], Child–Pugh Score [HR 2.240; (95% CI 1.188–4.224); *p* = 0.013], V-Stage [HR 2.420; (95% CI 1.187–4.936); *p* = 0.015], and resected segments ≥ 3 [HR 4.700; (95% 1.926–11.467); *p* = 0.001] as the major significant determinants of the 90-day mortality.

**Conclusion:**

Advanced patient age, pre-existing chronic renal insufficiency, Child–Pugh Score, extended hepatic resection, and vascular tumor involvement were identified as significant predictive factors of 90-day mortality. Proper patient selection and adjustment of treatment strategies could potentially reduce short-term mortality.

**Supplementary Information:**

The online version contains supplementary material available at 10.1007/s00423-024-03337-5.

## Introduction

Hepatocellular carcinoma (HCC) is the most common primary hepatic malignancy and a leading cause of cancer-related death worldwide [1, 2]. Hepatic resection (HR) and liver transplantation (LT) constitute the cornerstones of curative intended treatment even in advanced tumor stages [3, 4]. However, surgery-related mortality in HCC has been reported to range from 2% to 32%, depending on pre-existing cirrhosis and the extent of resection [5–7]. Morbidity rates after surgery range between 10% to almost 50% [8]. Advances in surgical techniques, significant improvements in perioperative care and proper patient selection in highly specialized and high-volume hepato-pancreato-biliary (HPB) centers have resulted in a remarkable reduction of perioperative mortality and morbidity [9, 10]. In order to avoid underestimation of postoperative mortality, the 90-day mortality rate has been proposed as a reliable measure of operative quality in hepatic surgery for malignancy since delayed mortality may not be registered within the first 30 postoperative days [11, 12]. Various clinical risk models and predictive scores of postoperative mortality in hepatic surgery have been described [13–18]. However their validity and accuracy in HCC must be questioned as these scores are constituted of a portfolio of arbitrarily chosen pre,-intra- and postoperative data/variables which were derived from heterogeneous study populations and tumor entities undergoing various types of parenchyma resection. The (model for end stage liver disease) MELD Score was therefore introduced to properly discriminate patients at risk of postoperative mortality in HCC patients with cirrhosis [19], whereas its predictive capacity diminished in non-cirrhotic patients [20].

Interestingly, only a few studies specifically analyzed the 90-day mortality outcome and its contributing parameters in elective HCC resection [21–24] resulting in a paucity of reliable and reproducible predictive mortality factors. Of note, three of these studies originated from Asian centers [21, 22, 24], one study only included preoperative clinical variables [24], while one study exclusively analyzed patients with major hepatectomy [22].

Hence, our primary goal was therefore to identify factors associated with 90-day mortality in a representative cohort of patients undergoing curative intent surgery for HCC. Identifying these factors will facilitate appropriate risk stratification and patient selection in order to minimize surgical morbidity and mortality by modifying adjustable parameters and considering alternative treatment options.

## Material and methods

All patients with HCC undergoing curative hepatic resection at the Department of General, Visceral, Thorax and Pediatric Surgery, University Hospital Duesseldorf, Germany between August 2004 and October 2021 were considered eligible from a prospectively maintained database. The exclusion criteria included patient age < 18 years, missing information regarding 90-day mortality, surgical exploration without parenchymal resection, and mixed typed tumors on final histology examination. Prior to the study initiation, the approval of the local ethics committee at the Heinrich-Heine-University Duesseldorf, Germany was granted (study-no.: 2021–1800- KFogU). All reported procedures and steps were in accordance with the principles of the latest version of the Declaration of Helsinki. All parts of this article were strictly subjected to the “Strengthening the Reporting of Observational Studies in Epidemiology” (STROBE) checklist for the reporting of observational Studies [25]. Data acquisition was organized and performed in four sections:

1. Preoperative: demographic and patient- related characteristics [age, gender, body mass index (BMI), ASA score (American Society of Anesthesiologists)], comorbidities, underlying hepatic disease and damage, baseline laboratory findings (including liver function tests, total blood count, renal parameters, albumin, α-fetoprotein, and hepatitis serology), MELD Score, Child–Pugh classification, preoperative imaging studies with tumor load and location, volumetric liver calculation in case of insufficient future liver remnant volume (FLRV), and alternative therapy concepts.
2. Intraoperative: type and extent of resection, biliary reconstruction, duration of surgery (min), number of transfused blood units, intraoperative complications.
3. Postoperative: morbidity including bile leakage, intra-abdominal abscess formation, cholangitis, sepsis, wound infection, notification of liver failure according to the International Study Group of Liver Surgery (ISGLS) criteria [26], and 90-day mortality as the primary endpoint.
4. Histology and tumor stage: total number of tumors, tumor diameter (mm), TNM classification based on Union internationale contre le cancer (UICC) 8th edition [27], tumor grading, distant metastasis, resection margin, perineural and lymphangio-invasion, nodal and vascular involvement.

The current available terminology of hepatectomy was applied to classify type and extent of resection [28]. Major hepatectomy was categorized as the resection of ≥ 3segments. Postoperative morbidity was defined and stratified based on the Clavien-Dindo classification [29]. The status of preoperative chronic renal insufficiency was determined according to the current nephrological guidelines with a glomerular filtration rate (GFR) < 60 ml/min per 1·73 m^2^ or markers of kidney damage of at least 3 months duration [30].

Each HCC patient was discussed in a multidisciplinary tumor board and the indication for surgical resection was confirmed by an expert panel of gastroenterologists, hepatobiliary surgeons, pathologists, radiotherapists and radiologists. Preoperative work-up included helical computed tomography (CT) scanning of the chest, abdomen, and pelvis. If necessary additional magnetic resonance imaging (MRI) scans of the liver were obtained for appropriate planning. Patients with extensive tumor burden and a prospective FLRV of < 30% were candidates for augmentation techniques using either portal venous embolization (PVE) or in situ split plus portal vein ligation (ISLT) at the discretion of the surgical team involved. Technical aspects and our institutional approach of in situ splitting of hepatic parenchyma has been discussed extensively [31, 32]. Moreover, we described the successful application of ISLT as a rescue procedure after insufficient growth with PVE [33]. Intraoperative hepatic ultrasound evaluation was routinely conducted to assess tumor extent and resectability, and to rule out undetected tumor nodules. Of note, the Pringle’s maneuver was not routinely applied for hepatic resection at our institution. Parenchyma dissection was done with the cavitron ultrasonic surgical aspirator (CUSA®; Valleylab, Boulder, Colorado, USA). Hepato-duodenal ligament lymphadenectomy was conducted for oncological and/or preparatory reasons and to visualize the hilar vascular and biliary anatomy in case of extended resections. After complex biliary reconstruction a decompressing T-Drain was inserted in some cases for optimal drainage and pressure reduction within the biliary tract. The retrieved specimen was subjected to precise histopathological examination. Tumor grading was performed according to Edmondson and Steiner [34]. Additionally, vascular and lymphangiovascular invasion, and the resection margins were determined by macroscopic and/or microscopic evaluation.

### Statistical analysis and variable selection

Statistical analysis was performed using the SPSS 25.0 software program (Statistical Package for Social Sciences; SPSS Inc., Chicago, IL, USA). To assess normal distribution of continuous data, the Shapiro–Wilk test was applied. Subsequently, the t-test was used for normal distributed data, while the Mann–Whitney U test was carried out for data that did not exhibit a normal distribution. Continuous data were expressed as median and standard deviation (SD). Categorical variables were summarized as frequencies (%) and compared using the chi-square or Fisher’s exact test. The 90-day mortality was defined as death within the 90-days interval from the initial hepatic resection. Patients were divided into two groups according to the occurrence of 90-day mortality. To identify potential risk factors for 90-day mortality based on pre- and intraoperative as well as histopathological parameters, Kaplan–Meier curves were generated and evaluated using the log-rank test. In addition, hazard ratios (HRs) with 95% confidence intervals (CIs) were estimated using a univariate Cox regression analysis. All relevant clinical and pathological variables with a *p*-value ≤ 0.1 were included into a multivariable Cox regression analysis. The forward stepwise selection was used to create a final model. Multiple imputation was used to replace missing values in our dataset. Variables with at least 20% missing values were excluded from the analysis. In all analyses, a *p*-value of < 0.05 indicated statistical significance.

## Results

### Patient characteristics and intraoperative data

A total of 196 (148 male/ 48 female) patients underwent curative HR for HCC at our department between 2004 and 2021. Preoperative patient characteristics, pathology reports and the intraoperative course are summarized in Table 1. The median age of the entire cohort was 68.5 years (range19-84 years). Ninety-three patients (47.45%) were seventy years or older. The majority of patients were classified as ASA III/IV (66.33%). Hepatitis B and C were evident in 44 (22.45%) and 58 patients (29.59%) respectively. Twenty- three patients (11.73%) had intermediate or advanced liver cirrhosis CHILD B and C while Child A cirrhosis was recorded in 150 (76.53%) patients. Thirty-nine patients (19.90%) had a history of chronic preoperative alcohol abuse. The most common comorbidities included cardio-pulmonary disease (56.63%), followed by diabetes mellitus type 2 (35.20%), and chronic renal insufficiency (14.80%). In 111 patients (56.63%) the MELD Score was at least as high as the median of 8. A single HCC lesion was noted in 116 patients (59.18%) whereas ≥ 2 lesions were recorded in 80 cases (40.82%). The rate of bilobular tumor burden was 29.59% (58 patients). Eighty-six patients (43.88%) underwent extended resections of ≥ 3 segments and complex biliary reconstructions were performed in 15 patients (7.65%). The median operative time in the entire cohort was 307 min (range 70–815 min) and a prolonged surgical procedure extending the median value was recorded in half of all patients (50%). Fifteen patients (7.65%) with a critical FLRV of less than 30%, required PVE or ISLT as hepatic augmentation techniques prior to extended resection. Rescue ISLT was necessary in 4 patients after unsuccessful PVE considering the insufficient volume gain. After histological examination, an advanced T-Stage (III/IV) was observed in 41 patients (20.92%). Thirty-two patients (16.33%) had high grade tumors (Grade III/IV). The rate of lymphangio-and vascular invasion was 5.10% and 17.35% respectively. Distant metastasis were observed in 5 patients (2.55%). In 35 patients (17.86%) R0-tumor clearance was achieved by a narrow resection margin (< 0.1 cm).

**Table 1:** Patient-tumor characteristics and operative data

Variables	All Patients (*n* = 196)	90-day mortality (*n* = 30)	90-day survival (*n* = 166)	*P*-Value
Age (years), [median ± SD] Age ≥ 70 years (n; %) Age < 70 years	68.5 ± 10.903 93 (47.45) 103 (52.55)	71.0 ± 9.633 20 (66.67) 10 (33.33)	68.0 ± 11.135 73 (43.98) 93 (56.02)	0.637 **0.022**
Sex (n; %)				
Male Female	148 (75.51) 48 (24.49)	26 (86.67) 4 (13.33)	122 (73.49) 44 (26.51)	0.123
BMI (kg/m^2^), [median ± SD] BMI ≥ 26.11 kg/m^2^ (n; %) BMI < 26.11 kg/m^2^	26.11 ± 4.321 98 (50.0) 98 (50.0)	25.66 ± 2.709 11 (36.67) 19 (63.33)	26.45 ± 4.54) 87 (52.41) 79 (47.59)	0.505 0.112
ASA Score (n; %)				0.085
ASA I/II ASA III/IV	66 (33.67) 130 (66.33)	6 (20.0) 24 (80.0)	60 (36.14) 106 (63.86)	
Laboratory parameters				
AST (U/l), [median ± SD] AST ≥ 52.5 (n; %) AST < 52.5 Bilirubin (mg/dl), [median ± SD] Bilirubin ≥ 0.71 (n; %) Bilirubin < 0.71 Hemoglobin (g/dl), [median ± SD] Hemoglobin ≥ 13.3 (n; %) Hemoglobin < 13.3 WBC (× 1000/µl), [median ± SD] WBC ≥ 6.13 (n; %) WBC < 6.13 Thrombocytes (× 1000/µl), [median ± SD] Thrombocytes ≥ 172.5 (n; %) Thrombocytes < 172.5	52.5 ± 77.137 98 (50.0) 98 (50.0) 0.71 ± 0.902 98 (50.0) 98 (50.0) 13.30 ± 2.070 100 (51.02) 96 (48.98) 6.13 ± 5.661 98 (50.0) 98 (50.0) 172.50 ± 92.154 98 (50.0) 98 (50.0)	78.36 ± 96.070 21 (70.0) 9 (30.0) 0.95 ± 1.398 18 (60.0) 12 (40.0) 12.90 ± 2.264 10 (33.33) 20 (66.67) 7.15 ± 4.058 19 (63.33) 11 (36.67) 182.35 ± 81.504 16 (53.33) 14 (46.67)	51.0 ± 72.048 77 (46.39) 89 (53.61) 0.71 ± 0.771 80 (48.19) 86 (51.81) 13.60 ± 1.991 90 (54.22) 76 (45.78) 6.10 ± 5.909 79 (47.59) 87 (52.41) 171.0 ± 94.053 82 (49.40) 84 (50.60)	**0.001** **0.017** 0.243 0.234 **0.011** **0.035** 0.060 0.112 0.812 0.692
Hepatitis A (n; %)				0.968
Yes No	20 (10.20) 176 (89.80)	3 (10.0) 27 (90.0)	149 (89.76) 17 (10.24)	
Hepatitis B (n; %)				0.184
Yes No	44 (22.45) 152 (77.55)	4 (13.33) 26 (86.67)	40 (24.10) 126 (75.90)	
Hepatitis C (n; %)				0.356
Yes No	58 (29.59) 138 (70.41)	11 (36.7) 19 (63.33)	47 (28.31) 119 (71.69)	
CHILD–Pugh Score (n; %)				**0.002**
0 A B C	23 (11.73) 150 (76.53) 22 (11.22) 1 (0.51)	3 (10.0) 18 (60.0) 8 (26.67) 1 (3.33)	20 (12.05) 132 (79.52) 14 (8.43) 0 (0)	
Comorbidities (n; %)				
Cardiac				0.134
Yes No	68 (34.69) 128 (65.31)	14 (46.67) 16 (53.33)	54 (32.53) 112 (67.47)	
Pulmonary				0.780
Yes No	43 (21.94) 153 (78.06)	6 (20.0) 24 (80.0)	37 (22.29) 129 (77.71)	
Renal				**0.011**
Yes No	29 (14.80) 167 (85.20)	9 (30.0) 21 (70.0)	20 (12.05) 146 (87.95)	
Diabetes mellitus				0.311
Yes No	69 (35.20) 127 (64.80)	13 (43.33) 17 (56.67)	56 (33.73) 110 (66.27)	
Alcohol abuse (n; %)				0.328
Yes No	39 (19.90) 157 (80.10)	4 (13.33) 26 (86.67)	35 (21.08) 131 (78.92)	
MELD Score (median ± SD) MELD Score ≥ 8 (n; %) MELD Score < 8	8.0 ± 3.591 111 (56.63) 85 (43.37)	8.50 ± 6.083 21 (70.0) 9 (30.0)	8.0 ± 2.661 90 (54.22) 76 (45.78)	**0.011** 0.108
Tumor Diameter (mm), [median ± SD] Tumor Diameter ≥ 45 mm (n; %) Tumor Diameter < 45 mm	45.0 ± 49.861 101 (51.53) 95 (48.47)	57.50 ± 64.517 18 (60.0) 12 (40.0)	44.50 ± 45.899 83 (50.0) 83 (50.0)	0.075 0.313
Single lesion (n; %) Multiple lesions (n; %)	116 (59.18) 80 (40.82)	18 (60.0) 12 (40.0)	98 (59.04) 68 (40.96)	0.921
Unilobular lesion(s) (n; %) Bilobular lesions (n; %)	138 (70.419 58 (29.59)	15 (50.0) 15 (50.0)	123 (74.10) 43 (25.90)	**0.008**
Pathology (n; %)				
T-Stage				0.069
I/II III/IV	155 (79.08) 41 (20.92)	20 (66.67) 10 (33.33)	135 (81.33) 31 (18.67)	
M-Stage				**0.005**
M0 M1	191 (97.45) 5 (2.55)	27 (90.0) 3 (10.0)	164 (98.80) 2 (1.20)	
Grade				0.259
I/II III/IV	164 (83.67) 32 (16.33)	23 (76.67) 7 (23.33)	141 (84.94) 25 (15.06)	
L-Stage				**0.026**
L0 L1	186 (94.90) 10 (5.10)	26 (86.67) 4 (13.33)	160 (96.39) 6 (3.61)	
V-Stage				**0.002**
V0 V1	162 (82.65) 34 (17.35)	18 (60.0) 12 (40.0)	144 (86.75) 22 (13.25)	
UICC-Stage				**0.002**
I II III IV	115 (58.67) 46 (23.47) 29 (14.80) 6 (3.06)	12 (40.0) 9 (30.0) 5 (16.67) 4 (13.33)	103 (62.05) 37 (22.29) 24 (14.46) 2 (1.20)	
Resection margin				
R < 0.1 cm R > 0.1 cm R < 0.5 cm R > 0.5 cm	35 (17.86) 161 (82.14) 76 (38.78) 120 (61.22)	6 (20.0) 24 (80.0) 13 (43.33) 17 (56.67)	29 (17.47) 137 (82.53) 63 (37.95) 103 (62.05)	0.739 0.578
Operative data				
ISLT/PVE (n; %)				**0.006**
Yes No	15 (7.65) 181 (92.35)	6 (20.0) 24 (80.0)	9 (5.42) 157 (94.58)	
Resected segments (n), [median ± SD]	2.0 ± 1.367	3.0 ± 1.356	2.0 ± 1.334	**0.002**
Segments ≥ 3 (n; %)				** < 0.0001**
Yes No	86 (43.88) 110 (56.12)	22 (73.33) 8 (26.67)	64 (38.55) 102 (61.45)	
Biliary reconstruction (n; %)				**0.006**
Yes No	15 (7.65) 181 (92.35)	6 (20.0) 24 (80.0)	9 (5.42) 157 (94.58)	
T-Drain (n; %)				0.609
Yes No	39 (19.90) 157 (80.10)	7 (23.33) 23 (76.67)	32 (19.28) 134 (80.72)	
Intraoperative transfusion (n; %)				0.087
Yes No	77 (39.29) 119 (60.71)	16 (53.33) 14 (46.67)	61 (36.75) 105 (63.25)	
Operative time (min), [median ± SD] Operative time ≥ 307 min (n; %) Operative time < 307 min Blood units (BU), [median ± SD]	307.0 ± 141.025 98 (50.0) 98 (50.0) 0 ± 9.560	383.50 ± 199.478 21 (70.0) 9 (30.0) 1.0 ± 23.192	292.50 ± 118.381 77 (46.39) 89 (53.61) 0 ± 2.819	**0.001** **0.017** **0.016**

*ASA Score* American Society of Anesthesiologists, *AST* Aspartate Aminotransferase, *BMI* Body mass index, *ISLT/PVE* in situ split plus portal vein ligation/portal venous embolization, *MELD* Model of end stage liver disease, *UICC* Union internationale contre le cancer, *WBC* White Blood Cells

### Postoperative course

The postoperative course is depicted in Table 2. The most prevalent postoperative morbidity was ISGLS grade B/C liver failure (32.65%). Wound infections were observed in 24 patients (12.24%). Twenty-one patients suffered from bile leakage (10.71%). In addition, intra-abdominal abscess formation was noted in 8.16% of the cases. Other infectious complications included sepsis (11.73%), and cholangitis (7.65%). The overall rate of severe complications (CD ≥ 3a) was 40.31%. Thirty patients died within the 90-day time interval from surgical intervention accounting for a 90-day mortality rate of 15.30% as the primary endpoint. Interestingly, when patients were stratified by study years, the 90-day mortality rate decreased from 19.69% in the time interval 2004–2015 to 7.8% in patients undergoing HR between 2016 and 2021.

**Table 2:** Postoperative outcome

Postoperative outcome (*n*; %)	All Patients (*n* = 196)	90-day mortality (*n* = 30)	90-day survival (*n* = 166)	*P*-Value
Bile leakage				0.891
Yes No	21 (10.71) 175 (89.29)	3 (10.0) 27 (90.0)	18 (10.84) 148 (89.16)	
Intra-abdominal abscess				**0.001**
Yes No	16 (8.16) 180 (91.84)	7 (23.33) 23 (76.67)	9 (5.42) 157 (94.58)	
Cholangitis				** < 0.0001**
Yes No	15 (7.65) 181 (92.35)	8 (26.67) 22 (73.33)	7 (4.22) 159 (95.78)	
ISGLS B/C				** < 0.0001**
Yes No	64 (32.65) 132 (67.35)	26 (86.67) 4 (13.33)	38 (22.89) 128 (77.11)	
Wound infection				0.106
Yes No	24 (12.24) 172 (87.76)	1 (3.33) 29 (96.67)	23 (13.86) 143 (86.14)	
Sepsis				** < 0.0001**
Yes No	23 (11.73) 173 (88.27)	20 (66.67) 10 (33.33)	3 (1.81) 163 (98.19)	
Clavien-Dindo ≥ 3a				** < 0.0001**
Yes No	79 (40.31) 117 (59.69)	29 (96.67) 1 (3.33)	50 (30.12) 116 (69.88)	

*ISGLS* International Study Group of Liver Surgery

### Uni-and multivariate analyses of predictive factors of 90-days mortality

First, a univariate analysis was performed to identify pre- and intraoperative as well as histopathological parameters that are associated with 90-day mortality. All variables with a *p*-value ≤ 0.1 were then included into a multivariate regression analysis. Accordingly, the following parameters were included into the multivariate analysis: age ≥ 70 years, ASA score I/II vs. III/IV, AST ≥ 52.5 U/l, hemoglobin ≥ 13.3 g/dl, Child–Pugh Score, chronic renal insufficiency, uni- versus bilobular lesion(s), T-Stage I/II vs. III/IV, M-Stage, L-Stage, V-Stage, UICC Stage ISLT/PVE, segments ≥ 3 resection, biliary reconstruction, operative time ≥ 307 min, intraoperative transfusion (supplementary Table 1). Subsequently, the final multivariate analysis revealed age ≥ 70 years [HR 2.798; (95% CI 1.263–6.198); *p* = 0.011], chronic renal insufficiency [HR 3.673; (95% CI 1.598–8.443); *p* = 0.002], Child–Pugh Score [HR 2.240; (95% CI 1.188–4.224); *p* = 0.013], V-Stage [HR 2.420; (95% CI 1.187–4.936); *p* = 0.015], and segments ≥ 3 [HR 4.700; (95% 1.926–11.467); *p* = 0.001] as significant predictive factors associated with 90-day mortality (Table 3).

**Table 3:** Multivariate analysis of predictors of 90-day mortality

Multivariate Analysis		
Variables	HR (95% CI)	*P*-Value
Age ≥ 70 years	2.798 (1.263–6.198)	0.011
Chronic renal insufficiency	3.673 (1.598–8.443)	0.002
Child–Pugh Score	2.240 (1.188–4.224)	0.013
V-Stage	2.420 (1.187–4.936)	0.015
Segments ≥ 3	4.700 (1.926–11.467)	0.001

## Discussion

The results of our single institutional study with 196 included patients demonstrate that the 90-day mortality rate after HR for HCC is significantly associated with advanced patient age (≥ 70 years), preoperative existent chronic renal insufficiency, CHILD–Pugh Score, vascular tumor involvement, and major hepatectomy with ≥ 3 resected segments. We deliberately omitted all postoperative factors from our analysis, as the main focus relied on pathological and (modifiable) pre- and intraoperative variables that allow for proper risk stratification and thus patient-tailored therapy.

The overall 90-day mortality rate in our cohort was 15.30%, which is consistent with a previous western report [23] but notably higher than Asian studies [35–37]. Results from a large German database query [38] demonstrated a hospital mortality rate of 9.3% in HCC patients following resection which might be a potential underestimation of the mortality data within 90 days, given our 30-day mortality rate of 6.63%. A recently published meta-analysis revealed a weighted 90-day mortality rate of 4.2% (range 3%–5.4%) among 8474 included patients with a significant level of heterogeneity [39].

Hepatic resection is still regarded as an effective and potentially curative method in HCC treatment. With the aging population and demographic changes, the percentage of elderly patients with pre-existing liver disease including HCC considered for hepatectomy is increasing [40]. However, the role of HR in the elderly population is still controversial based on conflicting results [41, 42]. Besides, a universal definition of “elderly” has not been described and studies used different cut-off values to stratify for patient age [43]. It has been previously shown that older patients over 70 years with liver cirrhosis undergoing hepatic resection are at increased risk of an unfavorable short-term outcome [44].These results were even confirmed in non-cirrhotic elderly patients after ≥ 2 segment resections [45]. Another large scaled study with 27.094 patients from Japan also depicted advanced patient age (≥ 70 years) as a significant risk factor of postoperative mortality in HCC [41].

The potential explanations rely on the underlying fragility and impaired physiological reserve capacities in this patient subgroup, especially in response to major surgical trauma [46], pre-existing liver deterioration related to liver cirrhosis and hepatitis [47], and the higher incidence of relevant comorbidities [41]. Of note, in our study almost half of the patients (47.45%) were aged 70 and above. The rate of major hepatic resection in the elderly group was 47.31%.

Another significant determinant of 90-day mortality was pre-existing renal insufficiency. Chronic kidney disease was reported in 29 patients (14.80%). The mortality rate in this patient cohort was 31.03%. Indeed, in the literature the impact of chronic renal insufficiency on short-and long term survival after HCC resection has been discussed with differing results [48, 49]. A recent meta-analysis [50] revealed that chronic kidney disease was associated with higher rates of postoperative complications and decreased overall survival. In another study, Shirata et al. [49] could demonstrate similar 90-day mortality rates in patients with chronic renal disease and Child–Pugh A cirrhosis in comparison to patients without renal impairment. In our cohort, the majority patients with renal disease had liver cirrhosis Child–Pugh A (79.31%). The 90-day mortality rate in this subgroup of Child–Pugh A patients with renal insufficiency was 21.73%, in contrast to Shirata et al. [49], who reported a 1.9% mortality rate following HR. Noteworthy, the rate of major hepatectomy in chronic renal disease patients was higher in our study (31.03% versus 27%) as compared to Shirata et al. [49] and a considerable proportion of our patients in this subgroup were classified ASA III/IV (75.86%). In the current study, the Child–Pugh classification was identified as a predictive factor of 90-day mortality. This is in line with a previously published work from Singapore analyzing 244 HCC patients [21]. The Child–Pugh score is a well-established clinical tool based on laboratory and clinical findings which helps to assess the severity of liver dysfunction and to predict postoperative outcome in hepatic surgery [51]. However, due to several limitations [52], other models such as the albumin–bilirubin (ALBI) score have demonstrated more accuracy in predicting overall survival in HCC resection [53]. In a large multicenter study, Beradi and colleagues [54] evaluated the 90-day mortality rate following 253 mostly minor hepatic resections in Child-B cirrhotic patients. The 90-day mortality rate of the entire cohort was 4.3% while a significant difference in 90-day mortality rate was observed depending on the extent of surgical resection (minor resection 3.3% versus major resection 10.3%; *p* = 0.04). In another European single center study [55] the in-hospital mortality rate of patients receiving HR for HCC stratified by Child–Pugh A, and B-C classification was 4.7% and 21.3% respectively (*p* = 0.0003).

The in-hospital mortality rate of the 23 included Child–Pugh B-C in our cohort was 13.04%, whereas the 90-day mortality rate increased to 39%. These numbers again highlight the importance of evaluating 90-day mortality instead of in-hospital and/or 30-day mortality to avoid underestimation and underreporting of the “true” operative related deaths [12, 56].

Of note, the rate of 11.22% Child–Pugh B patients in our study was higher than in the above mentioned studies of Beradi et al. (6.9%) [54] and Lei et al. (8.2%) [21]. The extent and type of resection has been proposed as a significant predictive factor and has subsequently been incorporated into risk scoring tools of in-hospital [57] and 90-day mortality [15, 23]. In contrast, a recent meta-analysis with 43 included studies could not demonstrate short-term survival benefits although long-term survival was significantly influenced by the resection strategy [58]. In our study the 90-day mortality rate of 86 patients undergoing major hepatectomy (≥ 3 segments) was 25.58% in comparison to just 7.27% in the group with minor resections (*p* < 0.0001). It is important to outline that this observation may be confounded by the remnant liver volume and the underlying liver function/disease as the majority of patients (88.27%) had various degrees of liver cirrhosis. Vascular infiltration negatively affects long-term survival in many studies [55, 59–61]. Interestingly, our study highlighted the presence of vascular tumor infiltration as a predictive factor of 90-day mortality. Of note, the rate of vascular tumor involvement included hepatic vein (HV), portal vein (PV), and inferior vena cava (IVC) infiltration rates of 12.24%, 2.04%, and 1.53% respectively. The high mortality rate of this subgroup is potentially linked to the greater proportion of extended hepatectomies (67.64%) with respect to anatomical and oncological considerations.

The presented results here must be interpreted taking into account the included study population and the proportion of minor and major resections performed. Over the years, we have followed a more liberal operative strategy with radical resections, even in older and comorbid patients, which might be an explanation of the overall higher mortality rate in the entire cohort compared to other reports. Noteworthy, we have observed a decline in the 90-day mortality rate from 19.69% to 7.8% since 2016. The same observation of improved outcome data was also reported by other studies [10, 62]. This trend in our cohort is largely attributed to careful patient selection for surgery, continuous technical modifications with less aggressive parenchyma resection, introduction of hepatobiliary scintigraphy, multidisciplinary approaches, and adjustment of modifiable pre-operative parameters. In fact, the rate of major hepatectomies in our department has fallen by almost 20% since 2016.

Our study has some important shortcomings with regard to its retrospective study design and the relatively small sample size. All data and results were derived from a single-western institution over an almost 20-years study period and are potentially not applicable to Asian cohorts with lower operative mortality rates. Additionally, technical refinements and changes in perioperative management and practice, which were not considered in our analysis, may have influenced the patient outcome after HR. Potential selection bias due to personal preferences and institutional guidelines, as well as missing value bias can further reduce the validity of the presented results. Furthermore, evolving non-surgical treatment modalities are not adequately addressed in this study setting. Finally, our data and results must be interpreted in the context of a natural evolution and improved learning curve in hepatic surgery from a European institution. Therefore, they could be an orientation and aid for comparable Western centers to adapt their surgical treatment strategy accordingly.

## Conclusion

Advanced patient age, pre-existing chronic renal insufficiency, Child–Pugh Score, extended hepatic resection, and vascular tumor involvement were identified as significant predictive factors of 90-day mortality following elective HR in HCC. Hence, proper patient selection and adjustment of modifiable pre-and intraoperative parameters could reduce the 90-day mortality rate as a surrogate marker of surgical quality and safety. Larger scaled and multi-institutional studies with comparable patient cohorts are needed to further validate the presented results.

### Supplementary Information

Below is the link to the electronic supplementary material.

Supplementary file1 (DOCX 17 KB)

## Data Availability

All the used and/or analyzed datasets in this study are available from the corresponding author on reasonable request.
