# Supplementary material for: Predictive factors of 90-day mortality after curative hepatic resection for hepatocellular carcinoma: a western single-center observational study
Source: Langenbecks Arch Surg. 2024 May 3;409(1):149. doi: 10.1007/s00423-024-03337-5 (PMC11065924; doi:10.1007/s00423-024-03337-5)
Supplement: Supplementary file 1 — Supplementary file1 (DOCX 17 KB) [file 423_2024_3337_MOESM1_ESM.docx]

Table Suppl. 1 Univariate analysis of predictors of 90-day mortality

| **Univariate Analysis** | | |
| --- | --- | --- |
| **Variables** | **HR (95% CI**) | **P-Value** |
| **Age ≥ 70 years** | 2.381 (1.115-5.088) | **0.021** |
| Sex (male vs. female) | 2.186 (0.763-6.263) | 0.135 |
| BMI ≥ 26.11 kg/m² | 0.556 (0.265-1.170) | 0.116 |
| **ASA score I/II vs. III/IV** | 2.144 (0.876-5.245) | **0,087** |
| **AST ≥ 52.5 U/l** | 2.476 (1.134-5.406) | **0,018** |
| Bilirubin ≥ 0.71 mg/dl | 1.530 (0.737-3.177) | 0.249 |
| **Hemoglobin ≥ 13.3 g/dl** | 0.464 (0.217-0.992) | **0.042** |
| Thrombocytes ≥ 172.5 (x1000/µl) | 1.156 (0.564-2.368) | 0.692 |
| WBC ≥ 6.13 (x1000/µl) | 1.796 (0.854-3.774) | 0.117 |
| Hepatitis A | 1.042 (0.316-3.436) | 0.946 |
| Hepatitis B | 0.519 (0.181-1.487) | 0.213 |
| Hepatitis C | 1.399 (0.666-2.940) | 0.373 |
| **Child-Pugh Score** | 2.296 (1.222-4.313) | **0.006** |
| Comorbidity |  |  |
| Cardiac | 1.723 (0.841-3.531) | 0.132 |
| Pulmonary | 0.859 (0.351-2.100) | 0.738 |
| **Renal** | 2.662 (1.219-5.815) | **0.010** |
| Diabetes mellitus | 1.459 (0.709-3.004) | 0.301 |
| Alcohol abuse | 0.605 (0.211-1.733) | 0.343 |
| MELD Score ≥ 8 | 1.794 (0.821-3.917) | 0.136 |
| Tumor diameter ≥ 45 mm | 1.447 (0.697-3.003) | 0.318 |
| Single vs. multiple lesion(s) | 0.965 (0.465-2.004) | 0.924 |
| **Uni vs. bilobular lesion(s)** | 2.548 (1.246-5.214) | **0.008** |
| **T-Stage I/II vs. III/IV** | 1.979 (0.926-4.227) | **0.072** |
| **M-Stage** | 5.020 (1.521-16.570) | **0.003** |
| Grade I/II vs. III/IV | 1.563 (0.671-3.643) | 0.296 |
| **L-Stage** | 3.100 (1.081-8.887) | **0.026** |
| **V-Stage** | 2.587 (1.460-4.583) | **0.002** |
| R < 0.1 cm vs. > 0.1 cm | 1.167 (0.477-2.855) | 0.734 |
| R < 0.5 cm vs. > 0.5 cm | 0.806 (0.391-1.659) | 0.556 |
| **UICC-Stage** | 1.686 (1.177-2.416) | **0.001** |
| **ISLT/PVE** | 3.369 (1.377-8.246) | **0.005** |
| **Segments ≥ 3** | 3.946 (1.756-8.865) | **<0.0001** |
| **Biliary reconstruction** | 3.230 (1.320-7.906) | **0.006** |
| T-Drain | 1.228 (0.527-2.863) | 0.633 |
| **Operative time ≥ 307 min** | 2.535 (1.161-5.535) | **0.015** |
| **Intraoperative transfusion** | 1.904 (0.929-3.902) | **0.073** |
